# Longitudinal association between social capital and recovery from depression and anxiety in adolescence: evidence from three cities in South America

**DOI:** 10.1192/j.eurpsy.2026.10334

**Published:** 2026-07-31

**Authors:** J. B. Kirkbride, V. Jasinska, G. Hudson, the OLA study

**Affiliations:** UCL, London, United Kingdom

## Abstract

**Background:**

Symptoms of depression and anxiety typically first emerge in adolescence and young adulthood, but factors that promote recovery, particularly for those exposed to social adversity, are poorly understood. We investigated whether perceived social capital was associated with clinical remission and symptomatic improvement for depression and anxiety over two years in a cohort of 1,437 young people living in the most deprived parts of Buenos Aires (Argentina), Bogota (Colombia) and Lima (Peru) in South America.

**Method:**

All participants, aged 15-16 and 20-24 at baseline and who met PHQ-8 or GAD-7 cut-offs for depression and anxiety at baseline, were followed for up to 24 months for evidence of clinical remission (change in symptom scores below cut-offs) or symptom improvement (continuous change in symptom scores). Remission was modelled as a repeated measure binary outcome at 6, 12 and 24 months using generalized linear mixed modelling with inverse probability weighting to handle attrition. Symptomatic improvement was modelled as a continuous outcome in separate linear mixed models for depression and anxiety. Exposures were self-reported measures of cognitive social capital (trust, reciprocity, shared values) and structural social capital (group membership, social support) at baseline using the ASCAT tool.

**Results:**

1,337 participants (93.0%) had at least one wave of follow-up data, with 46.3% meeting cut-offs for recovery (n=619) at one or more waves up to 24 months. In complete case analyses (n=1220, 91.2%) cognitive social capital was associated with greater odds of remission in a univariable (OR: 1.69; 95%CI: 1.04-2.78), but not full multivariable model (OR: 1.23; 95%CI: 0.83-1.75) after adjustment for confounders. Cognitive social capital was associated with symptomatic improvement in depression (Beta: -0.71; 95%CI: -1.40, -0.03) but not anxiety (Beta: -0.34; 95%CI: -1.00, 0.31) in multivariable linear models. We observed no main effects of structural social capital, but found strong evidence (p=0.0003) that high structural social capital was associated with greater odds of remaining above clinical threshold scores for depression/anxiety for adolescents (15-16 year olds) (OR: 1.78; 95%CI: 1.07-2.96), but greater odds of clinical remission for 20-24 year olds (OR: 1.83; 95%CI: 1.12-3.03).

**Conclusions:**

Higher cognitive social capital predicted symptomatic improvement in depression amongst young people in Latin America. Structural social capital showed age-dependent effects on clinical remission. Together, these findings suggest that interventions that allow young people to foster meaningful community ties that facilitate trust, reciprocity, connectedness and positive support may be an effective public mental health tool for secondary prevention of depression in young people.

**Disclosure of Interest:**

None Declared

